# The Use of Telehealth for Psychological Counselling of Vulnerable Adult Patients With Rheumatic Diseases or Diabetes: Explorative Study Inspired by Participatory Design

**DOI:** 10.2196/30829

**Published:** 2022-03-21

**Authors:** Mette Juel Rothmann, Julie Drotner Mouritsen, Nanna Skov Ladefoged, Marie Nedergaard Jeppesen, Anna Sofie Lillevang, Helle Laustrup, Torkell Ellingsen

**Affiliations:** 1 Rheumatology Research Unit Odense University Hospital Odense Denmark; 2 Steno Diabetes Center Odense Odense University Hospital Odense Denmark; 3 Centre for Innovative Medical Technology Odense University Hospital Odense Denmark; 4 Department of Clinical Research University of Southern Denmark Odense Denmark

**Keywords:** telehealth, videoconferencing, app, co-production, co-creation, psychologist, psychology, rheumatic diseases, diabetes, mobile phone

## Abstract

**Background:**

Video consultation is increasingly used in different health care settings to reach patients. However, little is known about telehealth in psychological counselling for vulnerable patients with somatic and chronic conditions such as rheumatoid arthritis and diabetes.

**Objective:**

This study aimed to develop and pilot test a telepsychology module for inclusion in the app My Hospital (Mit Sygehus) to provide remote psychological counselling to vulnerable adults with either rheumatic diseases or diabetes.

**Methods:**

With inspiration from participatory design, the content of the telepsychology module was developed through user involvement and evaluated by individual interviews with patients and psychologists as well as questionnaires.

**Results:**

We developed a module with our patient partners that targeted patients with rheumatic diseases and diabetes in relation to the psychological challenges of living with chronic diseases. The module included information, tools, exercises, and videoconferencing. In total, 16 patients and 3 psychologists participated in the pilot test. Psychological counselling was described by 4 themes: “The good relation despite physical distance,” “The comfort of being at home,” “The pros of saving time on transport and energy,” and “A therapeutic alliance at a distance.”

**Conclusions:**

Psychological counselling in relation to somatic care can be provided by videoconferencing supported by web-based or mobile delivery of tailored information, tools, and exercises without compromising on the quality of care. To ensure a good alliance between the patient and psychologist, a first face-to-face meeting is important. The home location provided patients with a safe environment and increased accessibility and reduced travel time to the hospital.

## Introduction

Telehealth in rheumatic and diabetes care is increasingly used to reach patients in rural areas as well as to maximize patient care in terms of reduced travel time to hospital sites and the convenience of consulting with clinicians from the patients’ own homes [1-4]. Lately, the COVID-19 situation has changed the way health care services are being delivered, with an increase in the use of telehealth [5,6]. Hence, there is currently a momentum for telehealth globally [6], which is important to build on, as the uptake of telehealth (eg, videoconferencing) in clinical practice has been slower than expected because of factors such as immature technology or unwillingness of health care professionals to adopt the new service [7,8]. In general, research has shown that patients are satisfied with psychotherapy provided through telehealth [9-11]. Furthermore, telehealth and telemental health have been shown to be as effective as in-person care [12,13]. Thus, several studies have shown videoconferencing to be safe and to have the same (or better) clinical effect as traditional consultations. Among others, these studies included patients with diabetes, heart failure, cancer, depression, and posttraumatic stress disorder [8,10,12,14]. In addition, a review on telepsychology has shown that video or phone sessions are effective for the treatment of conditions such as depression and anxiety [15]. Despite the growing empirical support for telemental health and telepsychology with regard to efficacy and patient satisfaction, literature on telepsychology for patients with somatic chronic conditions, such as rheumatoid arthritis and diabetes, is sparse. The term telepsychology is defined as “the provision of psychological services using telecommunication technologies” [16].

It is well known that patients with rheumatic diseases, such as rheumatoid arthritis, face challenges because of symptoms including joint or muscle pain and fatigue [17] and that many aspects of daily life in patients with diabetes are also significantly impacted because of psychological challenges, depression, and anxiety [18,19]. These challenges as well as the risk of depression, anxiety, and disease-specific distress can result in significant health consequences because of reduced coping strategies and self-management. Hence, psychological support and treatment are important in the care of patients with rheumatic diseases and diabetes [20,21]. However, there might be barriers to seeking help, which include comorbidities, geographical and time constraints, limited personal resources, and individual concerns. Even though Denmark is a small country, distance to the hospital is an important factor for inequality in the availability of health care services. In the long run, distance will also affect the individual patient’s ability to receive treatment because of the development in the Danish health care system where many treatment options (eg, disease-specific psychological counselling) are grouped at national university hospitals (6 in Denmark). Also, finances can be a barrier if patients are referred for psychological care (self-payment) by their general practitioner. Finally, it is inevitable that health care systems must change the manner in which health services are delivered because of demographic changes in patient populations, that is, increased aging population and burden of diseases, and must ensure equal access to health care. Telepsychology might be a way to address these barriers. A literature search has revealed sparse knowledge on telepsychology, telehealth, and psychological counselling for patients with rheumatic diseases as part of their somatic treatment. However, web-based psychoeducational intervention for patients with fibromyalgia syndrome has been shown to be effective on psychological variables [22]. Similar results were seen for a web-based program for youth with juvenile idiopathic arthritis [23]. Finally, a web-based, tailored cognitive behavioral intervention for patients with rheumatoid arthritis and a psychological risk profile has shown a positive effect on psychological outcomes [24]. In the field of diabetes, studies have demonstrated the effect of a web-based self-help tool in improving patients’ psychological well-being [25]. Group-based sessions with a focus on fear of hypoglycemia delivered remotely through telemedicine to parents of young children with type 1 diabetes (1-6 years of age) [26] and an mHealth service with automated interactive voice response that monitors patients’ self-management, provides immediate problem-tailored support, and connects to clinicians and the appropriate family members for feedback have also shown to be effective [27]. Furthermore, web-based psychotherapy programs have been shown to be effective in reducing depressive symptoms in patients with both type 1 and 2 diabetes [28]. There is a lack of knowledge about the use and acceptability of telepsychology compared to usual practice (same-room sessions). However, existing literature underpins that web-based psychotherapy may be effective in providing psychological counselling remotely. One such tool for telepsychology is the Region of Southern Denmark’s app My Hospital (Mit Sygehus). Launched in 2014, My Hospital provides relevant information to patients and their relatives. It consists of modules for each department in the region’s hospitals. My Hospital is easily available and free of charge. My Hospital is available in the App Store and Google Play as well as in a web version [29]. In general, the tools in the app are available without the need for individual user login. However, a personal login is needed to use features of the app where personal information and data are collected, such as videoconferencing, to comply with data protection regulation (the personal login for the app is secured and encrypted).

Hence, the purpose of this study was to develop and pilot test a telepsychology module for inclusion in My Hospital to provide remote psychological counselling to vulnerable adult patients with either rheumatic diseases or diabetes.

## Methods

This study was inspired by participatory design where the idea is to engage users to innovate and develop technologies in collaboration with developers [30]. In a traditional participatory design project, the users would be engaged from the beginning and throughout the study to ensure that the technology meets the end user’s needs [31]. In our case, we did not follow the participatory design methodology strictly, but engaged a former patient to participate in the development of the telepsychology module along with a colleague specialized in communication, information technology (IT) specialists, clinicians, and researchers. After the development phase, the patients referred to psychological counselling were invited to participate in the project and pilot test the module, including videoconferencing, and provide input for adjustments or changes.

This study was inspired from hermeneutics, where the perspective has been to understand the participants’ lived experiences in relation to living with rheumatologic diseases and diabetes to develop a service that meets the patients’ needs [32].

### Sample and Context

The participants were treated, followed, and recruited at the Department of Rheumatology and Steno Diabetes Center Odense, Odense University Hospital, Denmark. Patients were referred by nurses or doctors at the outpatient clinic to the department’s psychologists because of psychological challenges arising from living with chronic rheumatic disease or diabetes. The sampling was purposive and patients aged ≥18 years with rheumatic diseases or diabetes were considered eligible; however, the psychologists assessed the burden of disease. Thus, patients excluded were those who did not have any device at home (computer or tablet) and those who were assessed by the psychologist to be too sick for inclusion in the study, such as patients with very severe psychological problems. Furthermore, 3 psychologists participated in the study. The psychologists were master-level counsellors: 1 was newly qualified, 1 had a few years of experience, and 1 had more than 5 years of clinical experience.

### Data Collection

**Figure 1:**
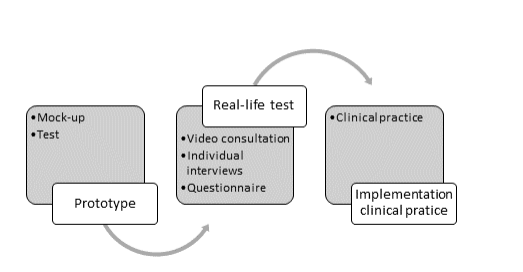
Data collection.

Data collection was divided into 2 processes (Figure 1) and performed between October 2018 and December 2019.

### Development of Prototype

**Figure 2:**
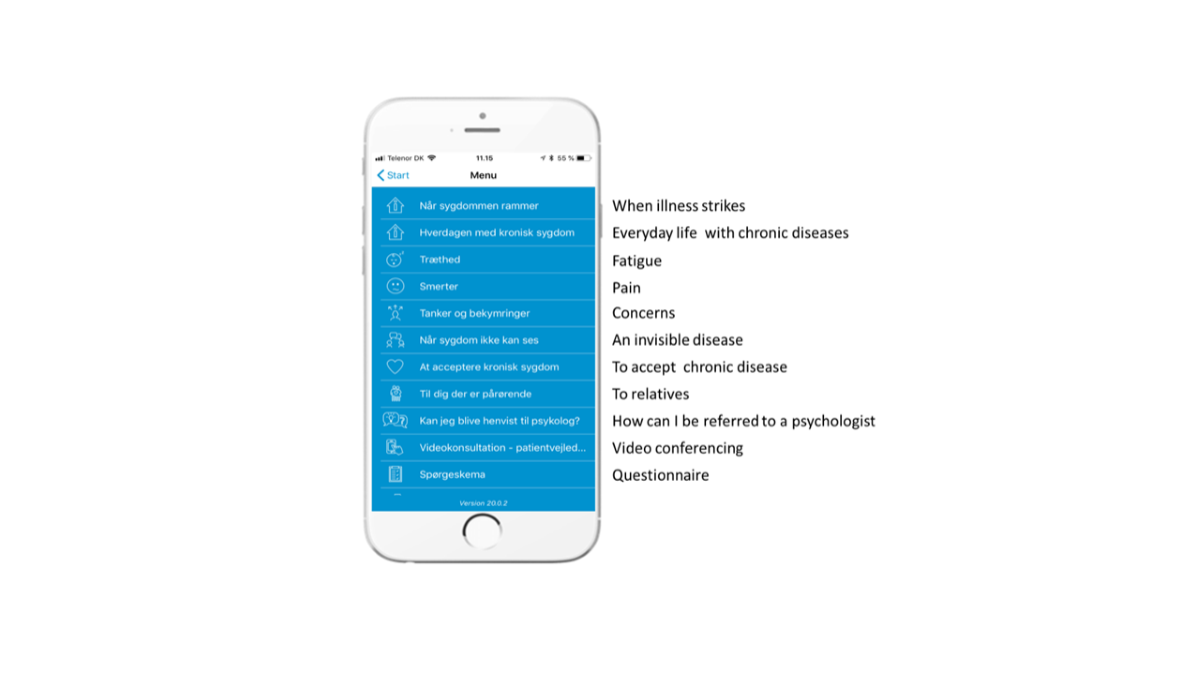
My Hospital.

First, a mock-up of the module including information and tools was developed based on clinical and specialist knowledge from the psychologist (unblended when published). The mock-up was discussed and re-designed in collaboration with a patient diagnosed with rheumatoid arthritis and a professional communicator specialized in patient involvement. The information and tools in the module were adjusted in an iterative process until they were accepted by all. Data were collected through track-change, notes, and meetings. Based on the feedback and changes, a prototype of the module was developed and included in My Hospital. The module included information on aspects such as everyday life with chronic disease, concerns, and acceptance, a worksheet with the cognitive behavioral model [33], and a description of mindfulness-based exercises. Thus, these could be used as basis for homework between the therapy sessions. Figure 2 shows a screenshot of My Hospital. The next step was test sessions, as videoconferencing has not been used for psychological counselling. My Hospital was updated to include videoconferencing. First, the technology for videoconferencing was tested, and login instructions were developed together with the patient, clinician, and IT specialist. The app and videoconference were integrated (partly) in the electronic patient record, allowing easy access for clinicians and integration to the booking system. The second author (JDM) conducted test sessions together with the patient to facilitate mutual learning throughout counselling. Psychological counselling was mainly based on cognitive behavioral therapy [33] and acceptance and commitment therapy [34]. Counselling consisted of conversation, exercises, and homework. The test sessions had a special focus on communication and the therapeutic alliance in relation to the counselling. Data on shared reflection and experiences with the technology were collected through notes.

### Real-life Test of the Service—the Intervention

Three psychologists from the departments were involved in the service, and 16 patients were invited to participate in the real-life test, in which usual counselling (individual planned sessions including 2-8 sessions) was converted into videoconferencing supported by the module in My Hospital and included information, tools, and exercises and homework between sessions. However, the first session was performed face-to-face at the hospital to ensure good relation as an important starting point for counselling. Furthermore, the psychologist was able to assess whether videoconferencing would be relevant and safe considering the severity of psychological problems.

The patient was given written instructions on how to access the app or web platform My Hospital and how to use My Hospital for videoconferencing as well as relevant contact information (phone numbers for the psychologists and IT support). The patient was verbally informed about the option of videoconferencing as an alternative to same-room counselling, necessary equipment (tablet or computer with a microphone and a camera, a well-functioning internet connection), a plan B in case of technical issues or dissatisfaction with the videoconferencing format (telephone counselling or same-room counselling), and the option of trying out videoconferencing with IT support before the first videoconference with the psychologist.

Results from the first phase showed that necessary equipment included a computer or a tablet with a camera, a microphone, and a strong internet connection. Smartphones were not recommended because of the small screen size and because they are hand-held devices. The study was based on “bring your own device” to minimize cost. Patients were automatically notified 15 minutes before a preplanned videoconference through a notification from My Hospital. This allowed the patient to “sign in” to the video platform so that the psychologist could start the videoconference. Data from the testing phase were collected through individual, semi-structured interviews with patients, and psychologists. MJR and JDM carried out the interviews. All interviews with patients were conducted by telephone or in the same room. The interviews varied between 30 and 60 minutes. All interviews were audio recorded. In addition, a short questionnaire was automatically sent to the patient by My Hospital after each videoconference. Furthermore, statistics from My Hospital showing the “click-rate” on different topics and indicating the topics that the patients read the most were collected. Finally, psychologists were asked to fill in log books on experiences with videoconference.

### Analysis

The data from the development phase were used to adjust and re-design the tool as well as to create preliminary guidelines for the service using a PDSA (Plan-Do-Study-Act) cycle approach [35]. The qualitative data from the second phase (real-life test) were analyzed by Braun and Clark’s text condensation [36]. First, we (MJR, MNJ) captured an overall impression of the data and extracted a preliminary set of main themes. Second, data were divided into meaningful topics relevant to the research question. Then, the topics were condensed and coded. Finally, the findings were synthesized, which involved a shift from condensation to descriptions and categories.

### Ethics 

According to Danish law, qualitative studies do not require approval from scientific ethics committees. However, this study was approved by the Danish Data Protection Agency (18/51158). The participants received both oral and written information before signing informed consent in compliance with the Helsinki Declaration. Data were stored and secure in a logged SharePoint, Region of Southern Denmark.

## Results

Results from the real-life pilot test are presented below. In total, 16 people agreed to participate in this study. Characteristics of the participants are shown in Table 1.

**Table 1:** Characteristics of participants (n=16).

Characteristic		Value		
**Gender, n**				
	Men		3	
	Women		13	
Age (years), range		25-59		
**Diagnosis, n**				
	Rheumatologic diseases		13	
	Diabetes		3	
**Number of sessions**				
	Mean	4.3		
	Range	2-10		
**Types of session, range**				
	In-person session	1-3		
	Video session	1-6		
	Telephone session	0-3		
**Distance to hospital, km**				
	Mean		53	
	Range		4-130	

A total of 45 videoconferences were conducted. All counselling sessions were of a shorter duration, varying from 1 to 10 consultations (including face-to-face and telephone consultations). Of the 16 patients, 6 (38%) agreed to participate in an individual interview. The remaining 10/16 (62%) patients were not able to participate because of their psychological state. Furthermore, individual interviews were conducted by 2 psychologists, and questionnaires were answered by patients after each videoconference.

### Results From Individual Interviews With Patients

The thematic analysis revealed the following 3 themes: “The good relation despite physical distance,” “The comfort of being at home,” and “The pros of saving time on transport and energy.”

#### The Good Relation Despite Physical Distance

In general, all participants had a good relationship with the psychologist despite the use of videoconferencing instead of same-room counselling. The foundation was established in a first same-room meeting, which all the participants described as very important to establish a trusting and safe environment. One woman said, “Initially, you do not know who you are going to meet, so it is nice to meet face-to-face” (Patient #4). However, one participant stressed that even though the relation was different from that in a same-room meeting, it was still good enough to ensure valuable counselling.

It’s different (video conferencing)...although I would like to give in…it’s just not the same as when we sit in the same room.

Patient #2

On the other hand, some (3/6) of the other participants stated that videoconferencing was as good as same-room meetings. Thus, the majority (4/6) of participants did not see videoconferencing as a barrier for the topics and issues to be discussed. One major reason for that was the professional skills of the psychologist in ensuring high-quality counselling through videoconferencing.

The psychologist is really good and she does well on video. She is present and the relationship is almost as good as when we meet face-to-face (in the same-room).

Patient #2

The participants felt that the psychologist not only had professional skills but also had special skills in relation to the use of technology and videoconferencing, that is, making eye contact, using appropriate tone of voice, pausing, and using tools.

Although the experience of videoconferencing use was positive, technical problems were described as an important barrier. The majority (5/6) of participants experienced different types of technical problems, all of which affected the session. However, the participants managed to continue the session by video, and they highlighted the importance of a “plan B” if there were problems such as knowing how to reconnect to video or switch to a telephone call.

#### The Comfort of Being at Home

In general, the participants had a very positive experience of the “home” sessions, as their home provides a safe environment. As 1 participant said, “I could cry...I had a secure environment, it's my home after all” (Patient #4). However, the participants stressed that there are certain challenges when therapy is provided at home. Sessions at home were new to everyone in the household and this required special attention. The participants had to find a suitable room for the conversation, and some (2/6) of the participants experienced lack of respect from family members. One participant said, “they just opened the door and came in…my husband…Oh, I just wanted to pick something up” (Patient #2). Lack of privacy was described by some (2/6) of the participants as something they had to talk about with their family members. On the other hand, one of the participants had her husband join the session. In general, the possible barriers at home were minor compared to the benefits of staying at home to “avoid transport and fatigue” (Patient #5).

#### The Pros of Saving Time on Transport and Energy

All participants described the benefit of not having to go to the hospital for counselling. Even for patients who live near the hospital, it could be stressful and take a lot of energy not only because of driving but also because finding a parking space can sometimes be impossible. Patients with rheumatic diseases (6/6), in particular, found it beneficial to stay at home, as their conditions were often associated with severe fatigue. As 1 patient said, “... when you do not have any energy left it doesn’t matter if you have to drive 1 or 50 kilometers” (Patient #4). The energy saved by staying at home was valued as important, and it enabled patients to participate in sessions and not cancel even on “bad days,” as the energy saved could be spent on counselling instead. One participant said, “So even though it had been a day with pain and lack of energy, I didn’t have to take the car, I just had to open my computer” (Patient #4). All participants described the benefit of saving time on transportation as saving energy, which enabled them to “use your energy on the right things” (Patient #6).

### Results From Individual Interviews With Psychologists

The interviews with psychologists revealed another meaningful theme: “A therapeutic alliance at a distance.

#### A Therapeutic Alliance at a Distance

In general, the psychologists were a little skeptical about the use of videoconferencing, and they questioned whether it was good enough and whether the therapeutic alliance could be built and maintained. They were worried about technical failure, as it would affect the alliance and leave patients on their own. On the other hand, the psychologists described how patients might benefit from the service, as many patients deselect same-room counselling because they already have too many appointments at the hospital and lack the personal resources to participate.

But there is also a unique opportunity to reach out to those who do not have the personal resources as it is takes too much energy and might have anxiety as well.

Psychologist #1

Thus, the benefits of videoconferencing compensated for the risk of technical problems. With time, the psychologists experienced that the therapeutic alliance was the same as that in same-room counselling. However, to ensure a good patient-psychologist alliance, both psychologists stated that the first session should always be face-to-face in the same room. Furthermore, they described how videoconferencing actually provided extra information, as the psychologist met the patient in their home and got a view of the patient’s daily life.

…it also affects the alliance as I can see a part of their life that I do not otherwise have access to, namely their home.

Psychologist #2

The benefits for patients were evident for the psychologist, as the home provided a safe environment for the patient. One of the psychologists said, “... I actually do not think there is a big difference (in the contact and alliance same-room versus video conferencing)…” [Psychologist #2].

However, it took courage to try videoconferencing because psychologists have to move away from the well-known consultation room to video. They described a feeling of losing control because of the unknown. In this new “room,” technology played a significant role because the risk of failure and technical breakdowns led to concerns among the psychologists, as it could prevent delivery of good, high-quality service. Especially in complex cases, the psychologist needed a sense of safety and in these cases, they were more concerned about the technology and distance to the patient.

...if I picked up something like a trauma, I was very aware of getting the patient to a same-room counselling next time.

Psychologist #1

Despite the concerns about the technology, the psychologists found that the videoconferences combined with the information and tools in the app were a good alternative to face-to-face sessions even though the technology might break down. Becoming familiar with the service was described as important, and the psychologists described how they had to develop new communications skills for videoconferencing to facilitate a good therapeutic alliance, for example, a plan B if the video failed.

...the patients also adapt fairly quickly (red. to video conferencing) when it has small delay in the sound or picture… you might interrupt each other…but it was okay as it did not impact on the conversation or the therapeutic alliance.

Psychologist #2

### Results From the Questionnaires

**Figure 3:**
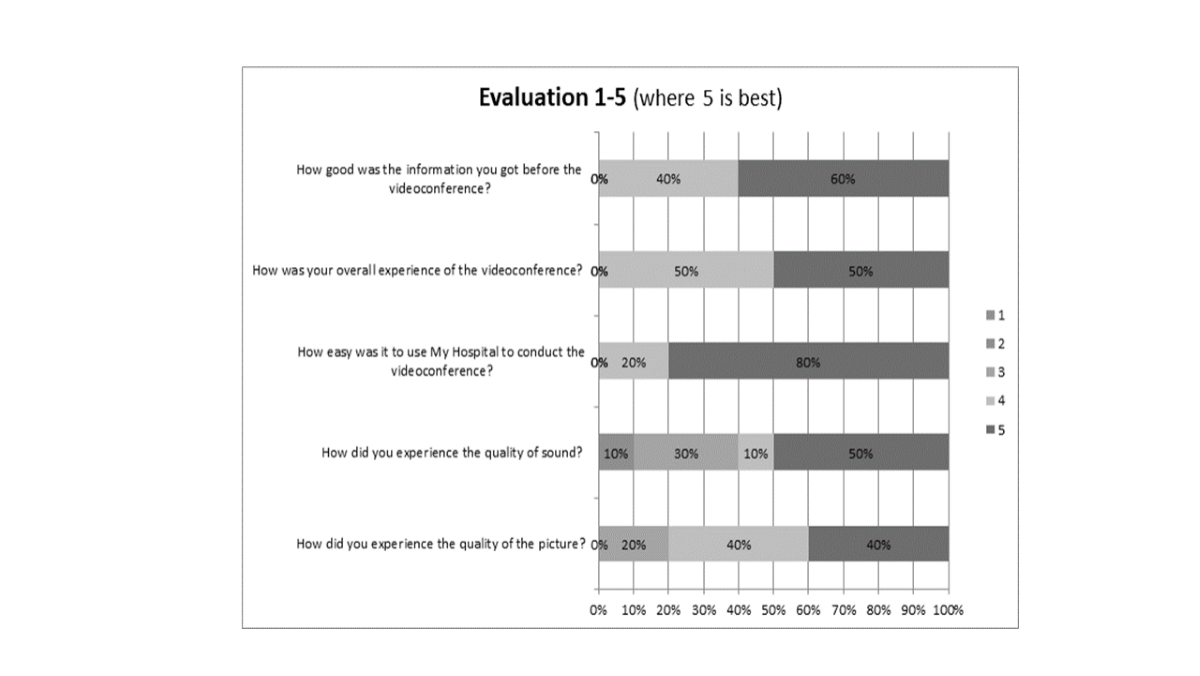
Evaluation of the service (n=16).

A brief questionnaire was released in My Hospital after each videoconference with the purpose of obtaining the patient’s immediate assessment of the counselling. A total of 16 patients completed the questionnaire. Data showed that the most common reason for choosing a virtual session again was “saving time on transport” followed by “Physical challenges e.g. parking, stairs.” The questionnaire also evaluated the information, accessibility, and quality of the virtual session. The results are shown in Figure 3.

Overall, videoconferencing was associated with a high degree of satisfaction with respect to information about the videoconferencing, the overall experience, the use of My Hospital, and the quality of sound and picture. However, data showed that there is room for improvement, as 6/16 (38%) participants rated sound quality as average or poor and 3/16 (19%) participants rated the quality of the picture as average. 

### Data From the App

The telepsychology module including information and tools in the form of text, videos, audio files, pictures, exercises, and videoconferencing was included in My Hospital. Once patients had been assigned to a treatment pathway in My Hospital, they could see an overview of topics related to the psychosocial challenges that can arise from living with a chronic disease. Data from My Hospital showed that topics such as “fatigue,” “pain,” “when illness strikes,” and “when illness is invisible” had the most views.

## Discussion

### Principal Findings

This study aimed at developing and pilot testing a telepsychology module for inclusion in My Hospital to provide remote psychological counselling to vulnerable adult patients with either rheumatic diseases or diabetes and psychological problems. The findings revealed that telepsychology and psychological counselling in relation to somatic care can be provided by videoconferencing and supported by tailored information, tools, and exercises.

To meet the needs of patients, we co-created a module in My Hospital together with a patient, a psychologist, a staff member specialized in patient communication, and IT experts to ensure inclusion of relevant knowledge from all participants [37]. The module contained information on aspects including everyday life with chronic disease, concerns, and acceptance, a worksheet with the cognitive behavioral model [33], and a description of acceptance and commitment therapy–based mindfulness exercises [34]. In line with previous studies using web-based tools [22-25,28,38,39] we found the module to be feasible and accepted by the patients. However, the module did differ from that in previous studies in that our module was designed to support the psychological counselling provided by videoconferencing. Hence, feedback on worksheets and exercises was given by the psychologist during counselling and not as interactive self-help [25], email messaging service [24,39], or phone calls [23]. Our study revealed that the patients found psychological counselling provided by videoconferencing to be a good alternative to same-room counselling at the hospital. This is supported by the findings of prior research evaluating telephone versus videoconferencing [40] and videoconferencing versus same-room–delivered therapy [41]. However, our study sample was relatively small, and it cannot be concluded whether videoconferencing is appropriate for all patients. Thus, the psychologists assessed some of the patients to be too vulnerable and sick for videoconferencing for reasons such as trauma. There is a need for evidence that can help us define specific populations who may most benefit from telepsychology, including videoconferencing and web-based counselling, and who may not. However, there is no evidence that counselling provided by videoconferencing can be harmful, and in general, it seems that patients benefit from the use of videoconferencing and web-based tools [2,42,43]. Still, existing literature shows that clinicians’ willingness to use and acceptance of telehealth are important for implementation [7]. In line with prior research findings on cost, travel time, and flexibility [26,44,45], our findings highlight the importance of being at home and saving time and energy, which can be of great importance to vulnerable patients. However, Matsumoto and Barton [2] point out that the increasing use of telehealth might introduce inequality because of lack of access (high-speed internet and smartphones). On the other hand, one could argue that patients might not get access to counselling if clinicians do not adopt the new service because of barriers such as cost and travel time. To minimize some of the barriers in telehealth, it is also important that the patients are interested in the service, have adequate IT skills, and have access to the necessary equipment [42]. We found that the module included in My Hospital was easy to access and use from the user’s own device (PC, tablet, or smartphone). Smartphones worked well for the module targeting information on the psychological impact of chronic diseases, but PCs or tablets were recommended for the videoconferencing to support a good relation and the psychological alliance, as they provide better picture, sound, and view of body language. Today, internet access and mobile technologies are an integrated part of everyday life for a large proportion of the population in industrialized countries, including Denmark. According to the most recent data from Statistics Denmark (2020), 95% of the Danish population has internet access [46], 90% have smartphones, 60% of families have tablets, 88% have laptops, and 36% have static PCs [47] in their homes. Thus, there might be a small proportion of the population without access to telehealth services. This could be managed by hospitals through the offering of tablets on loan to patients who need it. Moving of the therapy room to the home was well accepted, as the home provided a safe environment. However, this changed the setting at home, as the patients and their families were unfamiliar with videoconferencing and did not respect or think of the need for privacy during the sessions. Our findings stress the need for articulating issues of privacy in the family and to ensure that the patients are able to find a private space at home for the sessions. In addition, our findings revealed that it is important to have a clear-cut plan B if the technology or internet connection fails, as it could leave the patient vulnerable. In line with findings from studies on telemental health [11] and internet-based cognitive behavioral therapy [48], this study found that videoconferencing facilitated the therapeutic alliance as well as same-room counselling.

### Strengths and Limitations

A limitation of this study is that it was a small-scale study. However, the aim of this study, like other small-scale qualitative studies, was to provide in-depth exploration of the phenomenon under investigation. Therefore, the intention of this study was to develop and investigate the potential of telepsychology, including web-based tools and videoconferencing, for psychological counselling of patients with either rheumatic diseases or diabetes. It has to be taken into account that only 16 patients participated in the test, and that only 6 (38%) of these accepted participation in an individual interview. However, it is important to acknowledge that this study included vulnerable patients with severe psychological challenges, so the low degree of participation in interviews was somewhat expected.

That said, we have provided rich descriptions of both the development of the module (eg, information, worksheets, exercises) and potential of videoconferencing. The analysis was conducted in collaboration with co-researchers to increase reliability. To warrant validity, quotes from the interviews were used to link to the participants’ original statements. Hopefully, this will allow the readers to judge whether the findings of this study are transferable to their own contexts.

### Conclusions

Telepsychology and psychological counselling in relation to somatic care can be provided by videoconferencing and supported by web-based or mobile-delivered tailored information and tools. However, to ensure a good patient-psychologist alliance, the first session should always be in the same room. With a well-established alliance, the patients’ homes provided a safe environment and enabled them to conserve their energy that could be used on the right things, which is of great importance especially to patients with rheumatic diseases and severe fatigue. In patients with diabetes, the benefits were accessibility and reduced travel time to the hospital.

## Abbreviations

IT: information technology
PDSA: Plan-Do-Study-Act
